# A track record of Au–Ag nanomelt generation during fluid-mineral interactions

**DOI:** 10.1038/s41598-023-35066-y

**Published:** 2023-05-16

**Authors:** Diego Domínguez-Carretero, José María González-Jiménez, Joaquín A. Proenza, Cristina Villanova-de-Benavent, Xavier Llovet, Antonio Garcia-Casco

**Affiliations:** 1 Departament de Mineralogia, Petrologia i Geologia Aplicada, Universitat de Barcelona, Facultat de Ciències de la Terra, Barcelona, Spain; 2 Instituto Andaluz de Ciencias de la Tierra (CSIC-UGR), Granada, Spain; 3 Institut de Nanociència i Nanotecnologia, IN2UB Facultat de Química, Universitat de Barcelona, Barcelona, Spain; 4 Centres Científics i Tecnològics, Universitat de Barcelona, Barcelona, Spain; 5 Departamento de Mineralogía y Petrología, Facultad de Ciencias, Universidad de Granada, Granada, Spain

**Keywords:** Economic geology, Mineralogy

## Abstract

Recent studies have reported the significant role of Au-bearing nanoparticles in the formation of hydrothermal gold deposits. Despite the ever-increasing understanding of the genesis and stability of Au-bearing nanoparticles, it is still unknown how they behave when exposed to hydrothermal fluids. Here, we study the nanostructural evolution of Au–Ag nanoparticles hosted within Co-rich diarsenides and sulfarsenides of a natural hydrothermal deposit. We use high-resolution transmission electron microscopy to provide a singular glimpse of the complete melting sequence of Au–Ag nanoparticles exposed to the hydrothermal fluid during coupled dissolution–precipitation reactions of their host minerals. The interaction of Au–Ag nanoparticles with hydrothermal fluids at temperatures (400–500 ºC) common to most hydrothermal gold deposits may promote melting and generation of Au–Ag nanomelts. This process has important implications in noble metal remobilization and accumulation during the formation of these deposits.

## Introduction

In recent years, an ever-increasing number of works have reported Au-(Ag)-bearing nanoparticles (NPs) in natural colloidal suspensions —e.g., solid NPs with charged surfaces dispersed in an electrolyte solution— from fossil hydrothermal systems^1–3^ to modern geothermal fluids and black smokers^4–6^. Measurement of fluid inclusions containing these NPs from some epithermal^7^ and orogenic gold deposits^8^ reveal extremely high Au contents (up to 6000 ppm) in agreement with experimental works (e.g., Liu et al.^9^) indicating that NPs suspensions can concentrate up to ~ 5000 times more gold than as dissolved species. These new observations reveal that fluids that precipitate hydrothermal ores may have a higher capability to transport and deposit metals as previously thought. However, the precise mechanism(s) and location(s) of formation of these NPs in the hydrothermal ore systems, and the distance over which they may be carried out by the aqueous fluids, are still unclear. In his pioneering work focusing on the bonanza-vein gold ore from the Sleeper deposit of Nevada (USA), Saunders^10^ suggested that Au NPs could co-precipitate with colloidal silica at deeper levels in the hydrothermal system and be mechanically transported upward by the hydraulic action of the ore-forming fluids. Subsequent studies by Saunders and Burke^11^ and Burke et al.^12^ on epithermal ores, based on scanning electron microscopy (SEM) and high-resolution transmission electron microscopy (HRTEM), confirmed this proposal. More recently, McLeish et al.^2^ have proposed that Au-rich NPs may form in hydrothermal solutions when Au is close to saturation in the aqueous fluid, and that colloidal transport of these NPs is made it possible by a system that is maintained in a non-equilibrium state, arguing that both nucleation rates (high) and growth rates (low) would preclude the NPs growth and crystal formation —e.g., the upper levels of the porphyry environment where greater solubility yet vigorous episodic mixing and boiling is still possible. In contrast, Petrella et al.^3^ have suggested that metal NPs may be stabilized by colloidal silica (acting as a barrier that prevents deposition, dissolution or further growth of NPs) in emulsion and transported with the aid of low-density carbonic phases over kilometers through the Earth’s upper crust.

A careful inspection of HRTEM images of Au-rich NPs, identified at mineral-fluid interfaces of ore deposits with a protracted history of hydrothermal activity, reveals a predominance of spheroidal (i.e., droplet-like) morphology of the inclusions (see for example Fig. 6b, c in Deditius et al.^13^ and Fig. 6a in Hastie et al.^14^). These observations raise the possibility that immiscible Au-rich nanomelts could precede Au-rich NPs formation in some hydrothermal ore systems. This is, in principle, consistent with the experimental results showing that nano-liquids may be precursors of NPs in both aqueous solutions^15^ and solid mineral matrices (e.g., Reich et al.^16^; Becker et al.^17^). In the former case, the generation of Au-rich nanomelts can be linked to destabilization of metal-bearing aqueous complexes upon changes of temperature, pressure, Eh or pH in the solutions as well as to adsorption–reduction mechanisms of growing mineral surfaces precipitating from, or interacting with, the hydrothermal fluid^13,14,18^. For the latter, formation of Au-rich nanomelts may be accounted for solid-state dissolution of pre-existing metal-rich NPs in mineral matrices as observed experimentally in in situ heating experiments of Au-rich NPs in sulfide matrices (i.e., arsenian pyrite; Reich et al.^16^; Becker et al.^17^). In the experiments carried out by Reich et al.^16^ Au NPs of < 4 nm in size melted at much lower temperatures (370 °C) than bulk Au (1064 °C), in agreement with the size-dependent melting curve constructed by Buffat and Borel^19^ by melting isolated gold particles.

Despite these investigations conducted to understand the behavior of Au-bearing NPs, it is still uncertain how these NPs behave and texturally change during the destabilization of their host mineral. To date, there are no experiments analyzing the thermal stability of Au-rich nanomaterials (i.e., nanoparticles and nanomelts) in which interacting fluids and solid minerals are both involved. Indeed, Hastie et al.^14^ has provided the unique natural evidence of the co-existence of a Au-rich nanomelt and Au-rich NPs at mineral-fluid interfaces originated during processes of coupled dissolution–precipitation. These authors observed a contrasted behavior of Au-rich NPs in both oxides and sulfides, with a critical role of melts of polymetallic nature (i.e., consisting of low melting point chalcophile elements (LMCE), such as Te, Bi or Pb), during the nanoscale transport and fixation of Au. However, these authors could not resolve critical questions such as (*sic*): (1) the nature of gold within precursor pyrite to understand whether there is a conversion of elemental Au to NPs or simply NP liberation during dissolution; (2) the relationship among gold NPs and other elements, NPs and minerals; and (3) the roles that temperature and particle size in the formation and aggregation of gold NPs, specifically for conditions relevant to ore deposit formation.

This contribution aims to answer these pending questions after a careful examination of a suite of Au–Ag NPs hosted within diarsenides and sulfarsenides from the ultramafic-hosted volcanogenic massive sulfide (UM-VMS) deposit of Lomas de Majana (Cuba; see Supplementary Material for geologic information). The singularity of these minerals is that they preserve textural evidence of dissolution–precipitation processes in which three distinctively different types of Au–Ag NPs are recognized. The comparison of our results with experimental data allowed us to propose a genetic model, in which the exposure of Au–Ag NPs to the action of the hydrothermal fluid during mineral-fluid interaction, which involve coupled dissolution–precipitation of the host, produces NPs melting and subsequent metal remobilization via Au–Ag nanomelts. Our results have important implications in the current understanding regarding the origin and evolution of Au-bearing NPs in the hydrothermal fluids responsible for the genesis of hydrothermal gold deposits.

## Results and discussion

### Au–Ag nanoparticles: types, textures and host minerals

**Figure 1:**
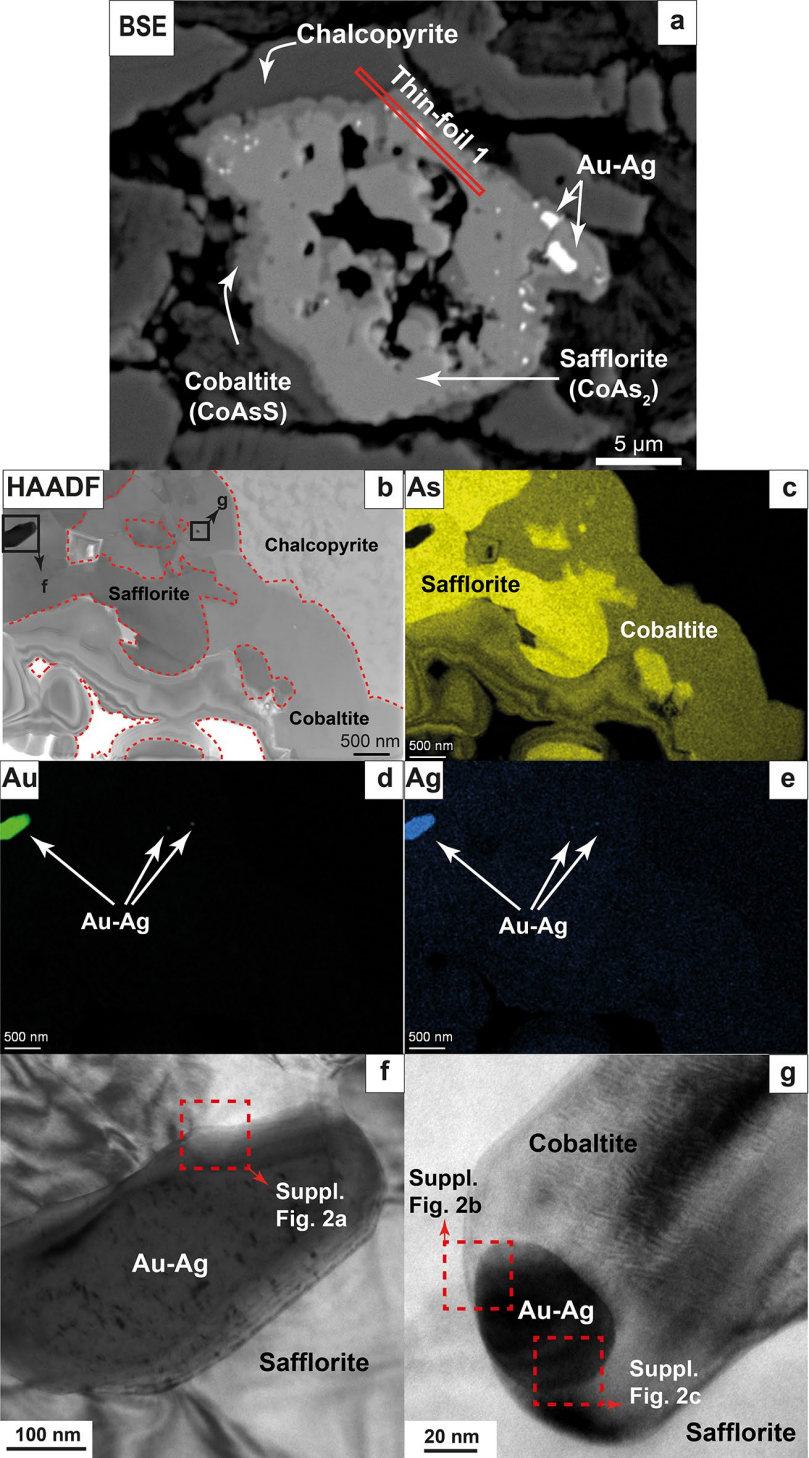
(**a**) Back-scattered electron (BSE) image of a safflorite grain with a cobaltite rim, surrounded by chalcopyrite. Several Au–Ag NPs can be observed within safflorite. The location of thin-foil 1 (obtained by FIB-SEM) is showed by a red rectangle. (**b**–**e**) High-angular annular dark field (HAADF) image and TEM-EDS maps for As, Au and Ag of the thin-foil. (**f**) HRTEM image of an euhedral Au–Ag NP. (**g**) HRTEM image of a droplet-like Au–Ag NP.

**Figure 2:**
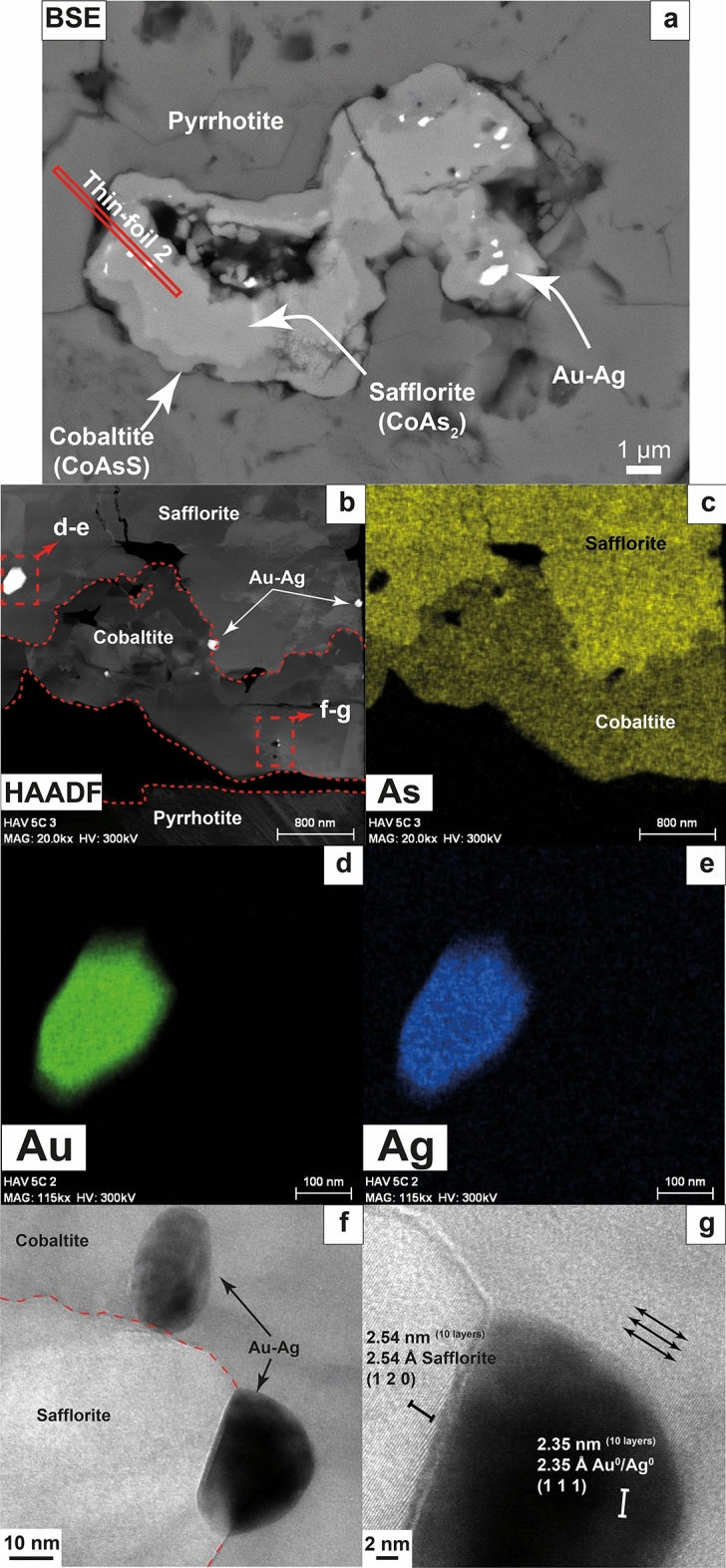
(**a**) BSE image of a safflorite grain rimmed by cobaltite and surrounded by pyrrhotite. Several Au–Ag NPs can be observed within safflorite. The location of thin-foil 2 (obtained by FIB-SEM) is showed by a red rectangle. (**b**, **c**) HAADF image and TEM-EDS map for As of the thin foil. (**d**, **e**) Detailed TEM-EDS maps for Au and Ag of an euhedral Au–Ag NP. (**f**) HRTEM image of a droplet-like Au–Ag NP and a partially euhedral, partially droplet-like Au–Ag NP found at the interface between safflorite and cobaltite. (**g**) Detailed HRTEM image showing the misorientation between the Au–Ag NP and safflorite crystal lattices.

Au–Ag particles with sizes varying from ~ 5 µm to 10 nm were found hosted within diarsenides [safflorite, CoAs_2_] and sulfarsenides [cobaltite, CoAsS] and their grain contacts (Fig. 1a and Fig. 2a). HRTEM examination of thin-foils cut by focused ion beam (FIB) from these textural locations (see analytical procedures in Supplementary Material; Fig. 1a and Fig. 2a) reveal three types of Au–Ag NPs: (1) euhedral particles up to 100–350 nm across, fully hosted within safflorite (Fig. 1b,f and Fig. 2b,d,e), (2) anhedral (droplet-like) particles up to 10–20 nm across Fig. 1g and Fig. 2f) fully hosted in cobaltite, and (3) subhedral particles ~ 20 nm across, located at the interface between the safflorite and cobaltite, with contrasting well-developed, straight outlines facing the safflorite and droplet-like facing the cobaltite (Fig. 2f, g). Transmission electron microscopy-energy dispersive spectrometry (TEM-EDS) maps show that all these three types of Au–Ag NPs exhibit internal chemical homogeneity (Fig. 1d, e and Fig. 2d, e), whereas HRTEM and selected area electron diffraction (SAED) patterns reveal a lack of crystallographic continuity with respect to their host matrices (Fig. 2g and Supplementary Fig. 2).

### In situ melting of Au–Ag nanoparticles

The interface between safflorite and cobaltite is irregular with embayment contacts (Fig. 1b, c, Fig. 2b, c, Supplementary Fig. 3 and Supplementary Fig. 4), while there are safflorite domains isolated within cobaltite matrix (Fig. 1b, c and Fig. 2b, c). These observations suggest a partial replacement of safflorite by cobaltite, very likely related to coupled dissolution–precipitation reactions. The partial dissolution and replacement of safflorite by cobaltite by hydrothermal fluids occurred at ca. 400–500 ºC and upon an increase in the *f*S_2_ and *f*O_2_^20,21^. In this scenario, some of the Au–Ag NPs originally hosted within the safflorite were partially or totally released, allowing interaction with the hydrothermal fluid. The change from euhedral faceted shapes (Fig. 1f and Fig. 2d, e) towards anhedral, rounded, droplet-like shapes (Fig. 1g and Fig. 2f) suggests a partial to complete melting of the particles being partially to completely exposed to the surrounding milieu as a result of heating by the infiltrating fluid. Soon after, the droplets of Au–Ag nanomelt were trapped and included within the growing cobaltite. Furthermore, the subhedral NP located at the interface between safflorite and cobaltite (Fig. 2f, g), which has euhedral shapes facing the safflorite and droplet-like shapes facing the cobaltite, indeed reflects an example of a partially exposed particle that melted only the volume that interacted with the hydrothermal fluid. The sequence we propose for a gradual shape transformation, and subsequent melting, of the exposed Au–Ag NPs is analogous to those observed in in situ heating experiments conducted on euhedral, metal NPs (i.e. Li et al.^22^; Li et al.^23^; Liu et al.^24^). In their experiments they observed that rising temperature produces the rearrangement of the metal NPs atomic structure, leading to the formation of melts at grain boundaries, such as at the grain surface. These melts, also called premelts, migrated through defects within the crystal lattice until, eventually, the NPs lose their euhedral, faceted shape and transform into molten nanospheres or nanodroplets (see Figs. 1, 2,3 in Li et al.^23^).

**Figure 3:**
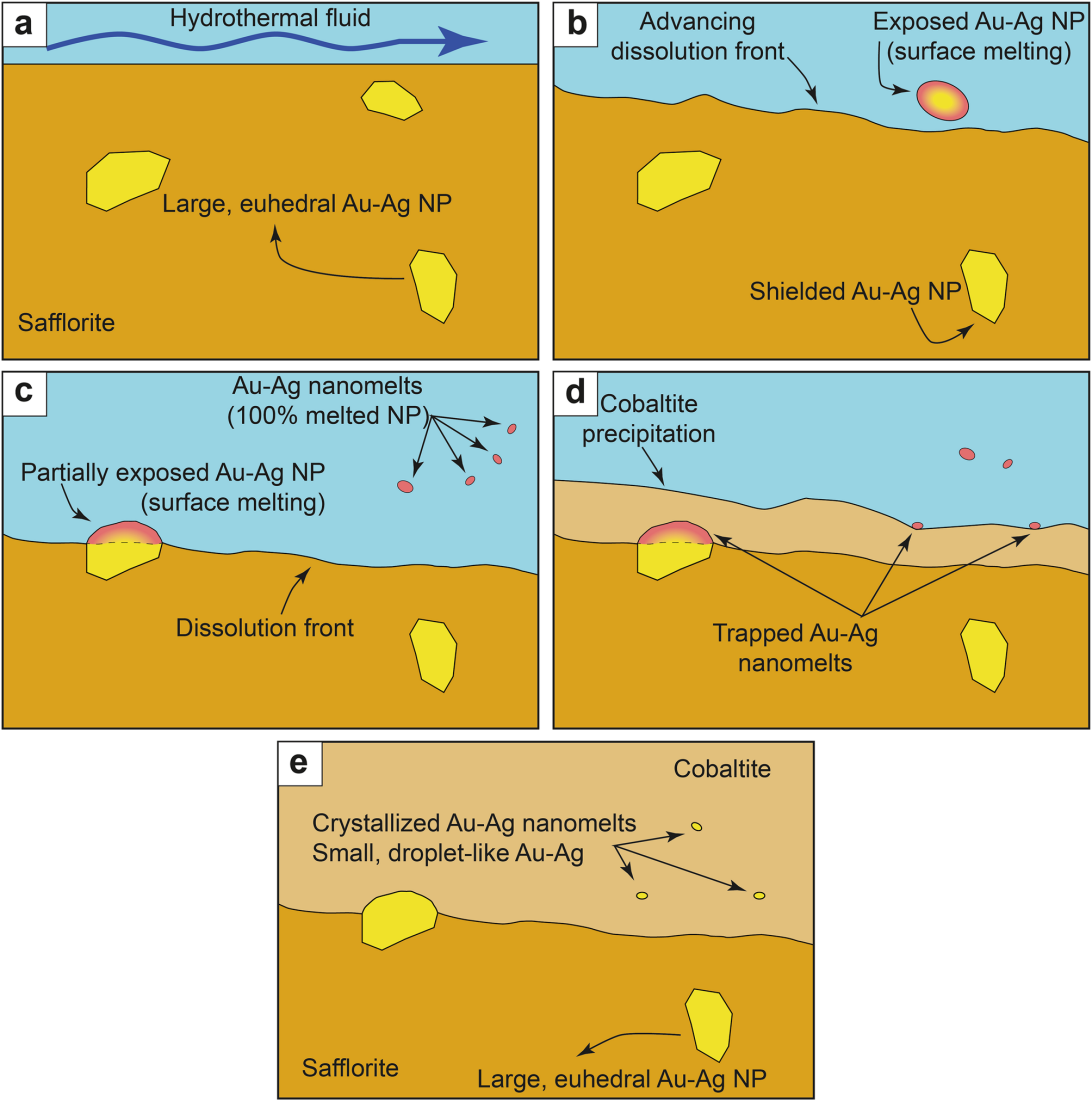
Genetic formation model of the three types of Au–Ag NPs. (**a**) Circulation of a fluid at 400–500 ºC and high *f*S_2_ and *f*O_2_ conditions produces the partial dissolution of safflorite. (**b**, **c**) Partial or total exposure to the fluid of the hosted Au–Ag NPs leads to formation of rounded morphologies and melting of their surface and, eventually, formation of Au–Ag nanomelts. (**d**) Precipitation of cobaltite, trapping small-sized droplets of Au–Ag nanomelts. (**e**) Cooling of the system produce the crystallization of trapped Au–Ag nanomelts into droplet-like Au–Ag NPs.

The euhedral Au–Ag NPs hosted by safflorite have larger diameters (100–350 nm) than the droplet-like Au–Ag NPs hosted within cobaltite (~ 20 nm). This difference in size is explained by the escape of Au–Ag nanomelts (or premelts) formed at the surface of the NPs prior to the complete melting of the whole Au–Ag NPs. Consequently, during the melting process of the NPs, these gradually shrink, and small batches of Au–Ag nanomelt are expelled from the original melting loci. Therefore, none of the smaller droplet-like Au–Ag NPs were melted in the place where they currently are. Conversely, the subhedral NP hosted at the safflorite-cobaltite contact that was in the process of being melted became trapped within the growing cobaltite before the Au–Ag nanomelt could escape from its original site. According to the experimental results by Lee et al.^25^, the heating of Ag NPs up to a few 100 s nm in size, submerged in water produces a significant size reduction of the NPs after the rise of temperature. Furthermore, Liu et al.^24^ performed *in-situ* TEM heating experiments to investigate the melting temperature of Ag NPs with diameters ranging from 60 to 120 nm, similar in size to our studied Au–Ag NPs, and observed the change of morphology upon temperature rise, losing their faceted shapes and transforming into rounded, droplet-like morphologies, indicative of melt formation, similar to our observations. These changes leading to NP melting took place at temperatures much lower (~ 375 ºC^24^) than the theoretical melting point for 60–120 nm sized particles (similar to the bulk melting point of Ag at 961 ºC^26^). Consequently, our observations, together with the results from the experiments by Liu et al.^24^, conflict with the proposed size-dependent melting curves of Au^19^ and Ag^26^, which predict that only NPs with sizes below 10 nm are able to melt within the estimated range of temperature of the studied hydrothermal system (400–500 ºC^20^). Regarding the size reduction of the NPs during melting, Lee et al.^25^ argued that NPs start melting at the surface, and the formed Ag nanomelt is driven away from its site of melting due to the repulsive forces acting at the interface between the NP and its corresponding melt. The process of separation of the Au–Ag nanomelt produced from the precursor NP could be effective considering the Au–Ag nanomelt formed at the surface of the melting NP as a real non-autonomous phase (Tauson et al.^27,28^). This latter phase should still preserve its crystal structure at the point of melting as its parental NP, while disordered soon after melt-production, comparably to those reported by Li et al.^23^ in their experiments. A similar process could take place in the studied samples considering that euhedral Au–Ag NPs are larger in size (100–350 nm) compared to the droplet-like, smaller (10–20 nm) Au–Ag NPs which crystallized from trapped Au–Ag nanomelts during the growth of cobaltite. Moreover, single-spot EDS analyses acquired by means of HRTEM show similar Au/Ag ratios in all types of NPs analyzed here (i.e., among large, euhedral NPs and small, droplet-like NPs; Supplementary Fig. 5), leading us to suggest that smaller, droplet-like Au–Ag NPs should derive from Au–Ag nanomelts physically separated from the larger, euhedral Au–Ag NPs.

## Conclusions

Partial dissolution of safflorite by a hydrothermal fluid with higher *f*S_2_ and *f*O_2_ and at temperatures between 400 and 500 ºC^20^ triggered partial to total melting of pre-existing euhedral Au–Ag NPs as they were partially to totally exposed to reactive hydrothermal fluids (Fig. 3a, b). Once liberated, these NPs melted due to thermal interaction with the surrounding hydrothermal fluid. The melting process is track recorded by a gradual change in the NPs morphologies from euhedral with faceted shapes towards anhedral, droplet-like shapes (Fig. 3b, c). Eventually, all the exposed volume of the NPs fully melted, producing smaller batches of Au–Ag nanomelts (Fig. 3c). Later, during the precipitation of cobaltite, small-sized droplets of Au–Ag nanomelts were trapped (Fig. 3d). Finally, during the cooling of the system, these trapped Au–Ag nanomelts crystallized into small (10–20 nm) droplet-like Au–Ag NPs (Fig. 3e).

Textural observations of the studied Au–Ag NPs reveal that their melting point is much lower than what the size-dependent melting curves of Au and Ag predict. Consequently, larger (10 s to 100 s nm) Au–Ag NPs in interaction with hydrothermal fluids are likely to melt and generate Au–Ag nanomelts at temperatures common to most hydrothermal systems. Therefore, metal remobilization via Au–Ag nanomelts is a phenomenon that must be considered when examining the genesis of hydrothermal gold deposits.

## Supplementary Information

Supplementary Information 1.

Supplementary Information 2.

## Data Availability

All data generated or analysed during this study are included in this published article [and its supplementary information files].

## References

[1] Saunders JA, Burke M, Brueseke ME (2020). Scanning-electron-microscope imaging of gold (electrum) nanoparticles in middle Miocene bonanza epithermal ores from northern Nevada, USA. Miner. Deposita.

[2] McLeish DF, Williams-Jones AE, Vasyukova OV, Clark JR, Board WS (2021). Colloidal transport and flocculation are the cause of the hyperenrichment of gold in nature. PNAS.

[3] Petrella L (2022). Nanoparticle suspensions from carbon-rich fluid make high-grade gold deposits. Nat. Commun..

[4] Hannington M, Hardardóttir V, Garbe-Schönberg D, Brown KL (2016). Gold enrichment in active geothermal systems by accumulating colloidal suspensions. Nat. Geosci..

[5] Gartman A (2018). Boiling-induced formation of colloidal gold in black smoker hydrothermal fluids. Geology.

[6] Hannington M, Garbe-Schönberg D (2019). Detection of gold nanoparticles in hydrothermal fluids. Econ. Geol..

[7] Banks DA, Bozkaya G, Bozkaya O (2019). Direct observation and measurement of Au and Ag in epithermal mineralizing fluids. Ore Geol. Rev..

[8] Prokofiev VY (2020). Exceptional concentrations of gold nanoparticles in 1,7 Ga Fluid Inclusions from the kola superdeep borehole northwest Russia. Sci. Rep..

[9] Liu W (2019). Colloidal gold in sulphur and citrate-bearing hydrothermal fluids: An experimental study. Ore Geol. Rev..

[10] Saunders JA (1990). Colloidal transport of gold and silica in epithermal precious-metal systems: Evidence from the Sleeper deposit. Nev. Geol..

[11] Saunders JA, Burke M (2017). Formation and aggregation of gold (electrum) nanoparticles in epithermal ores. Minerals.

[12] Burke M, Rakovan J, Krekeler MPS (2017). A study by electron microscopy of gold and associated minerals from round mountain Nevada. Ore Geol. Rev..

[13] Deditius AP (2011). Trace metal nanoparticles in pyrite. Ore Geol. Rev..

[14] Hastie ECG, Schindler M, Kontak DJ, Lafrance B (2021). Transport and coarsening of gold nanoparticles in an orogenic deposit by dissolution–reprecipitation and Ostwald ripening. Nat Commun. Earth Environ..

[15] Lee J, Yang J, Kwon SG, Hyeon T (2016). Nonclassical nucleation and growth of inorganic nanoparticles. Nat. Rev. Mater..

[16] Reich M (2006). Thermal behavior of metal nanoparticles in geologic materials. Geology.

[17] Becker, U., Reich, M., Biswas, S. Nanoparticle-host interactions in the natural systems, In *Nanoscopic Approaches in Earth and Planetary Sciences* (ed. Brenker, F.E., Jordan, G.) 52 p. (2010).

[18] González-Jiménez JM (2022). Polymetallic nanoparticles in pyrite from massive and stockwork ores of VMS deposits of the Iberian pyrite belt. Ore Geol. Rev..

[19] Buffat P, Borel JP (1976). Size effect on the melting temperature of gold particles. Phys. Rev. A.

[20] Domínguez-Carretero D (2022). Ultramafic-hosted volcanogenic massive sulfide deposits from Cuban ophiolites. J. S. Am. Earth Sci..

[21] Frost BR (1985). On the stability of sulfides, oxides, and native metals in serpentinite. J. Petrol..

[22] Li Y (2016). In situ study on atomic mechanism of melting and freezing of single bismuth nanoparticles. Nat. Commun..

[23] Li J, Wang Z, Deepak FL (2018). Direct atomic-scale observation of intermediate pathways of melting and crystallization in supported Bi nanoparticles. J. Phys. Chem. Lett..

[24] Liu M, Fu Q, Wang Z, Xie D, Wang Y (2019). Survey of transient process during melting of silver below the equilibrium melting point. J. Chem. Phys..

[25] Lee S, Phelan PE, Taylor RA, Prasher R, Dai L (2016). Low-temperature melting of silver nanoparticles in subcooled and saturated water. J. Heat Transfer.

[26] Luo W, Hu W, Xiao S (2008). Size effect on the thermodynamic properties of silver nanoparticles. J. Phys. Chem. C.

[27] Tauson VL, Lipko SV, Smagunov NV, Kravtsova RG (2018). Trace element partitioning dualism under mineral-fluid interaction: origin and geochemical significance. Minerals.

[28] Tauson V (2019). Distribution of “invisible” noble metals between pyrite and Arsenopyrite exemplified by minerals coexisting in orogenic au deposits of north-eastern Russia. Minerals.

[29] Iturralde-Vinent MA (2016). The geology of cuba: A brief overview and synthesis. GSA Today.

[30] Abdullin AA, Aniptov IA, Septov NS (1999). Allochthon displaced mineral deposits and their occurrence (in Russian). Geologica Kazakhstan.

[31] Llanes, A.I. et al. Petrología y mineralización de la asociación ofiolítica de Habana-Matanzas (Cuba Occidental). Memorias Geomin. 92–101 (2001).

[32] Cazañas, X. et al. Mapa Metalogénico de la República de Cuba a escala 1:250 000. Instituto de Geología y Paleontología, Centro Nacional de Información Geológica, p. 95 (2017).

[33] Llanes, A.I. et al. Informe final sobre los resultados del proyecto I+D 253 evaluación del potencial de Au endógeno en ofiolitas de Lomas de Majana, Salomón y Galindo, región Habana-Matanzas y II etapa. Reporte, Instituto de Geología y Paleontología de Cuba, 26p (2006).

[34] Llanes-Castro, A.I. Constitución y génesis de las ofiolitas de la región de Habana-Matanzas. Unpublished PhD. thesis, Universidad de Pinar del Río, p. 250 (2016).

