# Supplementary material for: A track record of Au–Ag nanomelt generation during fluid-mineral interactions
Source: Sci Rep. 2023 May 16;13:7895. doi: 10.1038/s41598-023-35066-y (PMC10188599; doi:10.1038/s41598-023-35066-y)
Supplement: Supplementary file 1 — Supplementary Information 1. [file 41598_2023_35066_MOESM1_ESM.docx]

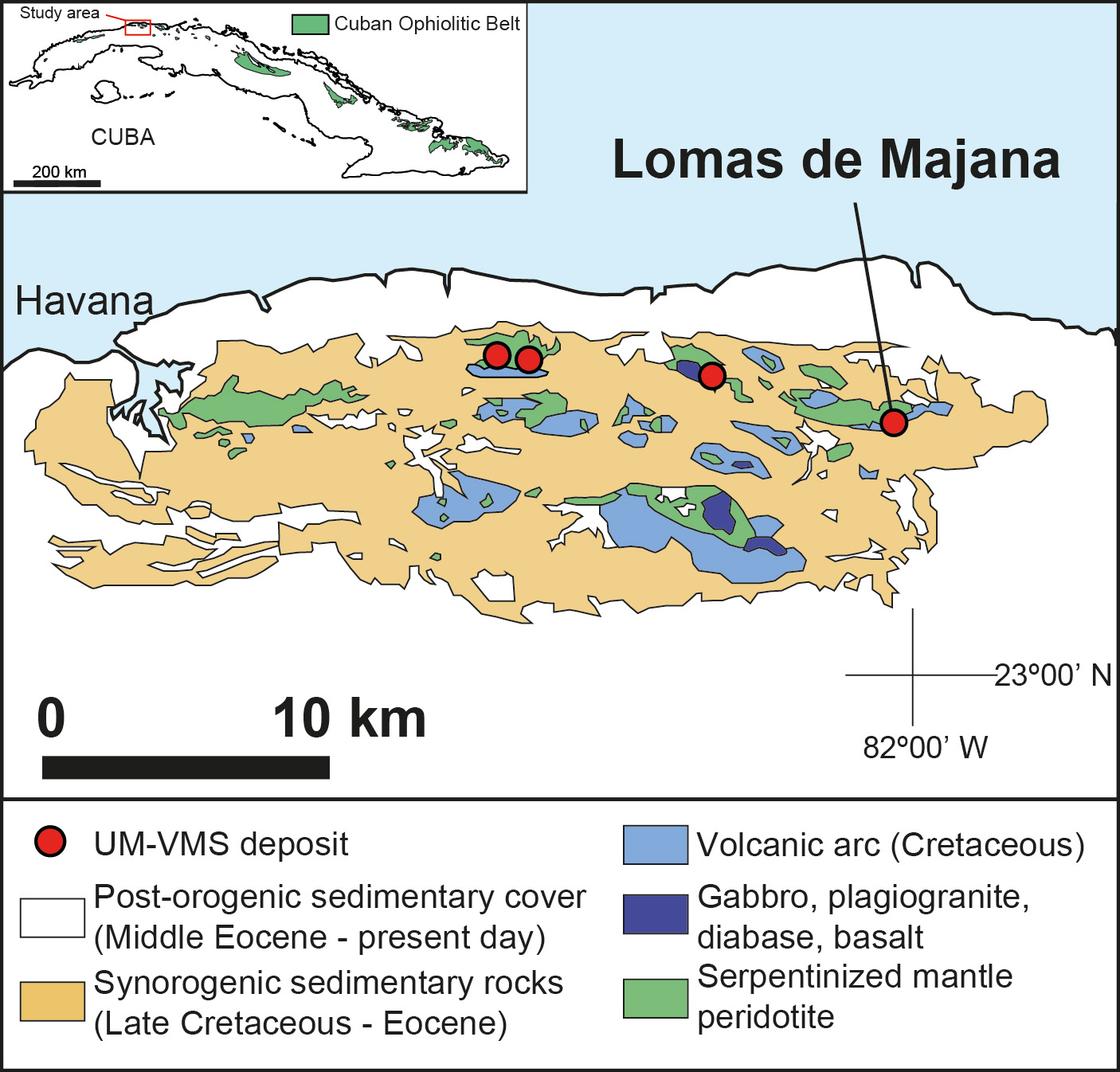


Supplementary Figure 1. Geological map of Havana-Matanzas with the location of the Lomas de Majana UM-VMS deposit. Modified from Llanes-Castro^34^ and Domínguez-Carretero et al.^20^.


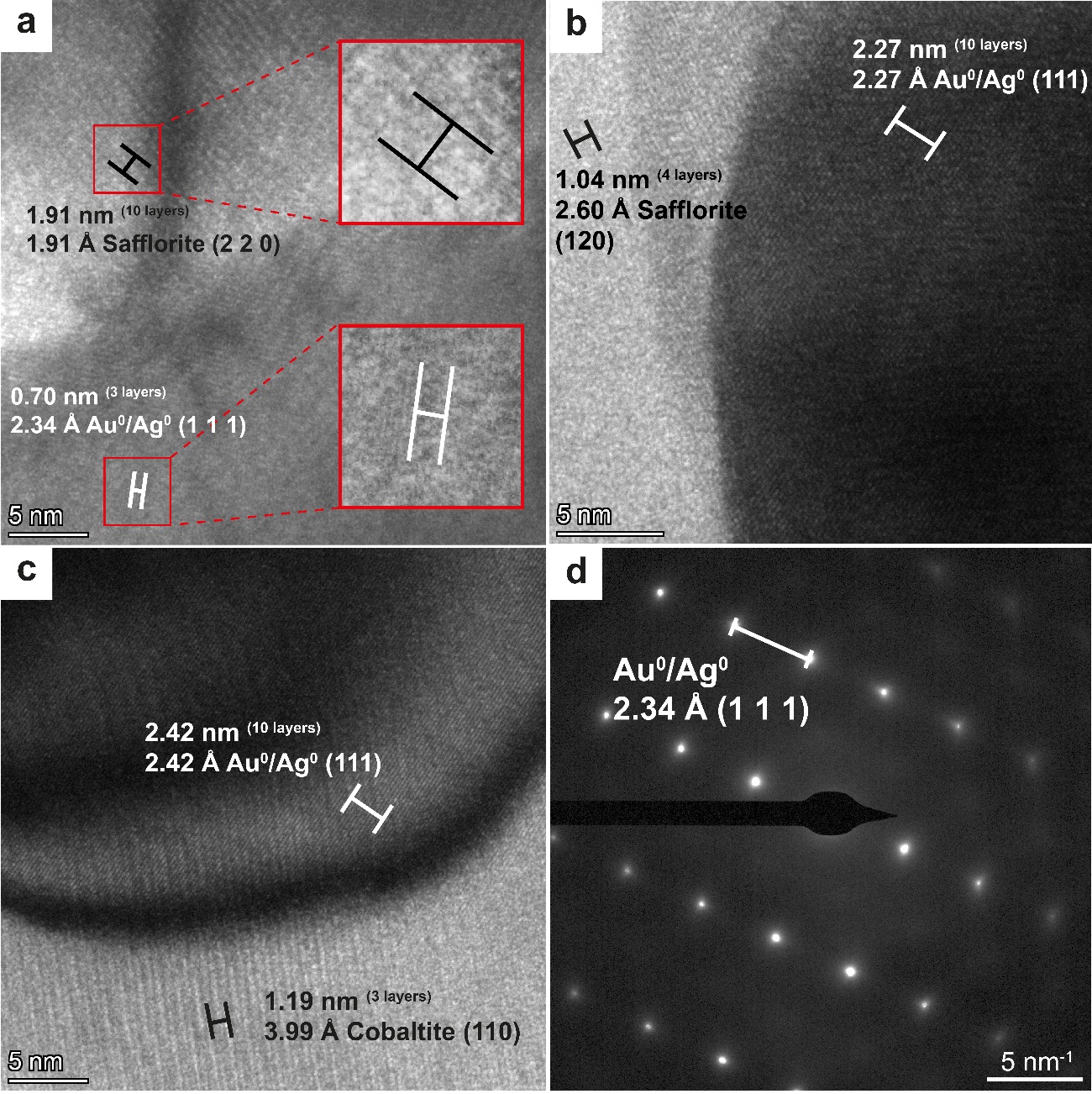


Supplementary Figure 2. a-c) HRTEM images showing the misorientation between the Au-Ag NPs lattices and their host mineral matrices. d) Selected Area Electron Diffraction (SAED) pattern corresponding to the euhedral Au-Ag NP in Fig. 2d-e.


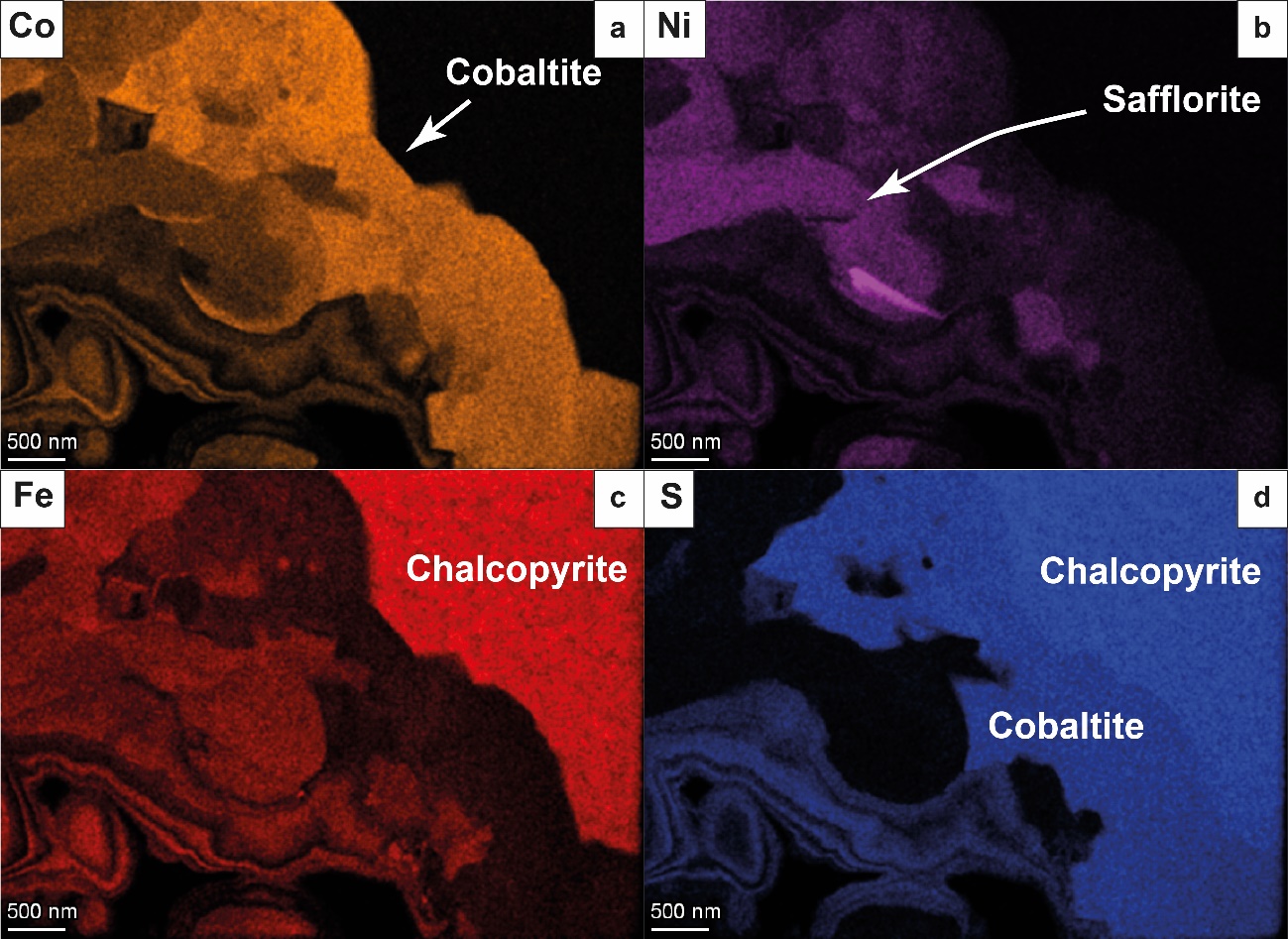


Supplementary Figure 3. TEM-EDS maps for Co, Ni, Fe and S of thin-foil 1.


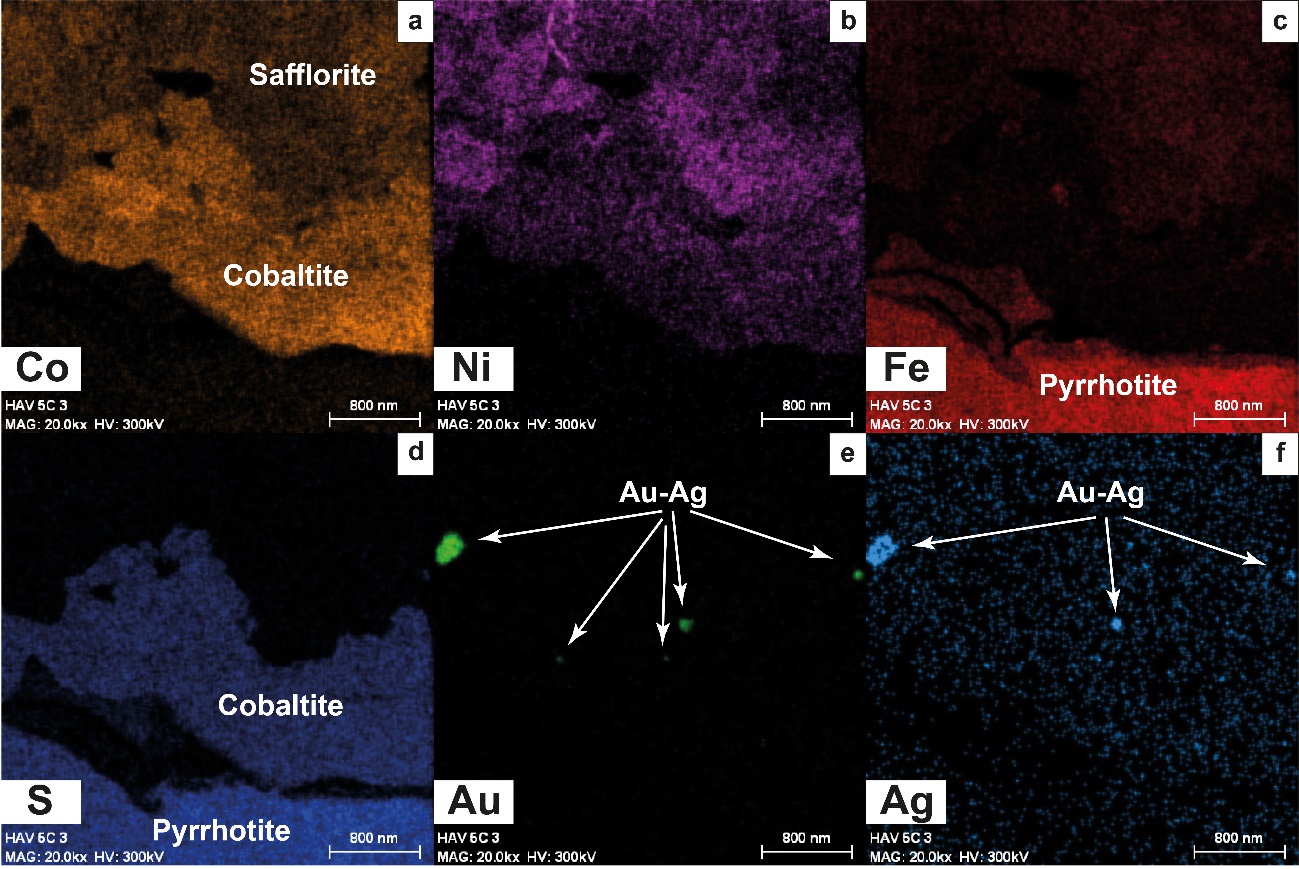


Supplementary Figure 4. TEM-EDS maps for Co, Ni, Fe, S, Au and Ag of thin-foil 2.


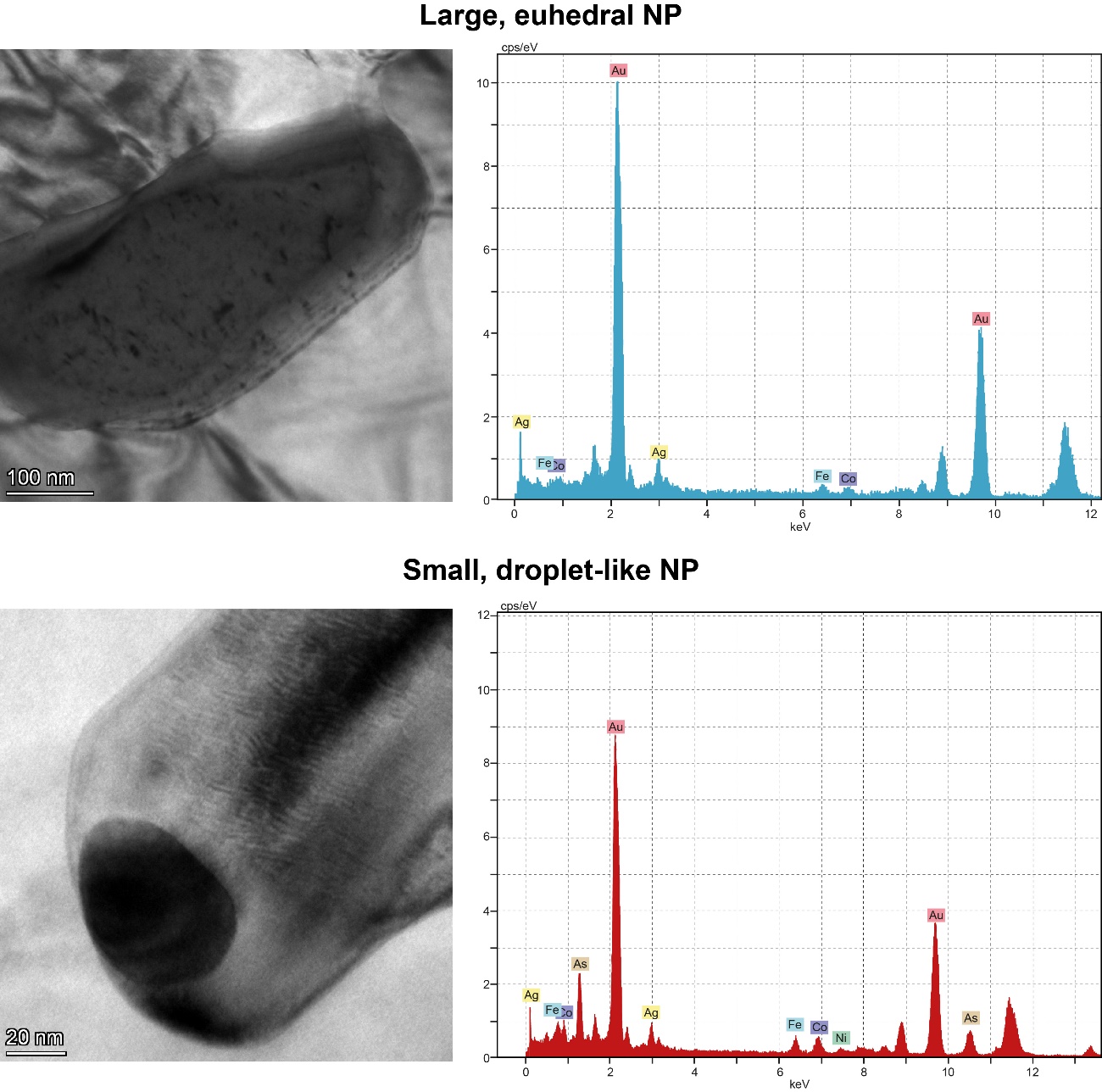
Supplementary Figure 5. EDS spectra of a large, euhedral NP and a small, droplet-like NP. Note how the Au and Ag peaks have comparable heights for both types of NPs, therefore denoting similar Au/Ag ratios for euhedral and droplet-like NPs. As peaks in the EDS spectrum shown below are due to sulfarsenide matrix fluorescence effect owing the much smaller size of the NPs.
