# Supplementary material for: A track record of Au–Ag nanomelt generation during fluid-mineral interactions
Source: Sci Rep. 2023 May 16;13:7895. doi: 10.1038/s41598-023-35066-y (PMC10188599; doi:10.1038/s41598-023-35066-y)
Supplement: Supplementary file 2 — Supplementary Information 2. [file 41598_2023_35066_MOESM2_ESM.pdf]

Supplementary material

Geological setting

The studied samples were collected in the Lomas de Majana UM-VMS deposit, within the Havana-Matanzas Ophiolitic Massif, which forms part of the ca. 1000 km long Cuban Ophiolitic Belt (Supplementary Fig.1; Iturralde-Vinent et al.,[27]and references therein). The Havana-Matanzas Ophiolitic Massif contains the only UM-VMS mining district throughout the Caribbean region, and seven different UM-VMS deposits have been described: Lomas de Majana, Salomón, Cruz Verde, Guanabo, Elena, Caridad and Vigilante[20,28-30]. The mineralization in the Lomas de Majana deposit is made of massive sulfide bodies (up to 50 m in length and 5 m in width) hosted within highly serpentinized harzburgites and dunites from the Moho transition zone (MTZ)[20,31]. The sulfide mineralization consists mostly of pyrrhotite, with lesser amounts of chalcopyrite, cubanite, Co-bearing pentlandite, Co-Ni-Fe diarsenides (safflorite-rammelsbergite-löllingite series), partially replaced at the rims by Co-Ni-Fe sulfarsenides (cobaltite-gersdorffite-arsenopyrite series), and electrum[20]. The mineralization was formed by reduced, metal-rich fluids circulating through serpentinized mantle peridotites exhumed via low-angle, detachment faults in an extensional fore-arc environment[20].

Methods

Focused ion beam (FIB)

Two electron transparent thin-foil samples were prepared and extracted using a dual beam FEI Thermo-Fisher Scientific Helios 650 focused ion beam scanning electron microscope (FIB-SEM) at the Laboratorio de Microscopías Avanzadas (LMA) at the Instituto de Nanociencia de Aragón (INA) – University of Zaragoza, Spain. The selected areas of interest were firstly covered by a thin strip (~ 300 nm) of C by focused electron beam deposition (FEBID), followed by a second strip of Pt (~ 1 µm). These two strips acted as protectors during the extraction process of the thin foils. The bulk material was first removed on both sides of the lamella by a rough Ga^{+} ion milling at 30 kV with 2.5 nA current and the subsequent polishing at 30 kV with 0.23 nA current. The final polishing step was completed by milling the samples at 5 kV with 68 pA current until electron transparency was achieved. An Everhart-Thornley SE detector was used to monitor the electron transparency of the samples. Once the electron transparency was obtained, the thin-foil samples were rapidly polished at 6 kV using a

low current of 10 pA to reduce amorphization until a final thin-foil thickness of ~100 nm was achieved. After that, the sample was undercut at 30 kV with a 2.5 nA current, lifted-out and transferred to a TEM grid using an OmniProbe nanomanipulator with a tungsten tip. Finally, ion-beam assisted Pt deposition was performed to weld the thin-foil samples to the tungsten tip, and subsequently to the TEM grid.

High-resolution transmission electron microscopy (HRTEM)

A combination of a Thermo Fisher Scientific TALOS F200X and a FEI Titan G2 TEMs equipped with Field Emission Gun (FEG) was used to analyze the thin-foils at the Centro de Instrumentation Científica at the University of Granada, Spain. Both the TALOS and the FEI Titan G2 microscopes are equipped with 4 energy dispersive spectrometers (EDX) X-ray detectors (FEI microanalysis Super X) and a high-angle annular dark-field (HAADF) detector. The FEI Titan G2 microscope has a spherical aberration correction at the objective lens.

Selected mineral areas of interest sampled within the thin-foil were imaged on the FEI Titan G2 microscope using a combination of HAADF imaging, to obtain Z high contrast images, and high-resolution transmission electron microscopy (HRTEM) imaging, to characterize the texture of the grains and to properly define the ordering of the mineral aggregates. All the images were treated using the Digital Micrograph® software Version 1.71.38, while maps were processed with the VELOX® software package. The FEI Titan G2 was operated at 300 kV and HRTEM images were acquired using a Gatan CCD Camera. The Thermo Fisher Scientific TALOS F200X TEM was employed for single-spot analytical electron microscopy (AEM) analysis as well as to collect compositional elemental mappings using a 200 kV accelerating voltage and image drift correction. Elemental maps were also processed using the VELOX® software.
